# Editorial

**Published:** 1870-02

**Authors:** 


### Editorial.

### MEDICAL ETHICS.

OMAHA, NEB., *Jany. 10th, 1870.*

N. S. DAVIS, M.D.:

*Dear Doctor:*—I desire an opinion from some physician, acknowledged by the fraternity at large, in this country, to be a man of eminent ability, and one of the lights of our profession. When a private pupil of the late Dr. Alden March, of Albany, I first learned particularly of you; and as I often heard him speak of your ability and attainments, in the highest and most complimentary terms; and as you have been the recipient of the most distinguished honors the profession of our favored land could bestow, and have stood foremost and in front among the organizers and leaders of our great National Association, I feel that your opinion, if not conclusive, would be entitled to the greatest weight and the most serious consideration.

We have, in this city, a regularly and duly organized medical society. It is a respectable association, and a deep interest is felt in it by its members. It contains within the pale of its membership, nearly all of the members of the regular profession residing in town; and in it is embodied nearly all the experience and ability of the profession in this place. In accordance with custom, we have established a bill of prices or charges for professional services; one, we believe, in keeping with the labor we have to perform and the demands made upon us by the public, and justly demanded by the dignity and importance of our noble calling.

Now, there are regular practitioners of medicine in this city who have been invited to become members of our Society, but have failed or neglected to do so; and they assign as a cause of such failure or neglect the following reason: that a membership in our Society would be pecuniarily detrimental to them; in other words, that by charging less for their services than our established rate of prices calls for, they hope to make inroads and encroachments upon our practice, and secure that patronage which would otherwise be ours. But when these gentlemen have under their treatment a difficult, serious case, they are quite ready to avail themselves, when a consultation is desired, of the advantages accruing from fellowship with members of our Society, who are, some of them, men of large experience; thus securing to themselves whatever of importance, weight, and prestige may attach to our organization. The code of medical ethics of the American Medical Association, I revere, as a patriot reveres the Constitution of our country; and I believe it is a safe, wise, and just policy for the faculty to strictly adhere to and scrupulously carry out its wholesome provisions. In consulting the Code, I find in Subdivision 1st, Art. 4th, which bears upon consultations, the following language:—"No intelligent regular practitioner, who is in good, moral, and professional standing in the place in which he resides, should be fastidiously excluded from fellowship, or his aid refused in consultation, when it is requested by the patient."

The question then arises: are these doctors in good professional standing and entitled to the benefits and advantages that may accrue from consultation with members of our Society? Are they consistent practitioners?

Medical societies are evidently instituted, not only to promote mutual improvement of the members, in the science and art of medicine and surgery, but also to protect the regular profession against the assaults, encroachments, and combinations of all forms of irregularity and charlatanism. Is there not embodied in the above case a species of irregularity? Shall we continue to consult with these physicians, if they refuse or neglect to become members of our Society, and continue to disregard our established rate of prices? Please answer at length, and greatly oblige. Yours truly,

GEO. TILDEN, M.D.

In answering the questions of our correspondent, we would refer to Article 8, of the Second General Division of the Code of Ethics, which is as follows:—

"Some general rules should be adopted by the faculty in every town or district relative to *pecuniary acknowledgments* from their patients; and it should be deemed a point of honor to adhere to these rules, with as much uniformity as varying circumstances will admit."

While we do not think that any physician should be discarded from consultations or professional recognition, *merely* because he does not become a member of a medical society, when one exists in the place of his residence; yet if his neglect to become a member is acknowledged to be from a desire to charge less than the generally established rate of charges in his locality, his position, certainly, cannot be regarded as an honorable one in the profession. And if it be clearly ascertained that he habitually charges patients, able to pay, less than the rates agreed upon, and actually charged by a large majority of his professional brethren in the same town, it should be deemed sufficient evidence of dishonorable conduct to justify his exclusion from consultations and professional recognition. It is for the interest of every practitioner that there should be some generally acknowledged rate of charges within the circle of his practice. It is only by such generally acknowledged rate that he can legally enforce his own claims against any one of his patients. While medical societies should be cautious in establishing their rates of charging or fee-bills, not to make them such as to create the impression in the public mind, that their organization is simply a combination for exacting unreasonable compensation for services; yet when a reasonable rate of charging has been actually adopted by a fair majority of the active and honorable practitioners of any town or district, every physician practising in such town or district should consider himself honorably bound to regulate his charges in accordance with it. If any should persist in refusing to do so, we do not see how they could be regarded as "practitioners in good standing" in the profession.

BOGUS DIPLOMAS.—In the January number of the EXAMINER we published a notice of a "collegate agency," in Milwaukee, Wisconsin, which proposed to furnish diplomas from medical colleges and other educational institutions, to such as needed them, simply as a matter of merchandise.

Yesterday, we received from another correspondent a card of a similar character, headed "Collegiate Agency," and signed "Dr. A. J. Hale, 214 Jacoby Street, Philadelphia." Accompanying the card are three letters written by Dr. Hale to one of his correspondents.

In the first, he offers to furnish the diploma conferring the degree of M.D. for $80, and of A.M. for $50. In the second letter he puts down the price of the medical degree to $50, and urges his supposed applicant to send in his full name to be entered in the diploma at once.

The third letter is as follows:—

PHILADELPHIA, PA., *Nov. 23d, 1869.*

DR. ———:—

*Dear Sir:*—Yours of 19th inst. to hand. Did not think it necessary to specify all the institutions conferring degrees. If you wish to apply to an Allopathic, I refer you to the Dean of one institution, "American University of Philadelphia," Prof. John Buchanan, Dean, 225 North 12th Street. If you wish to apply to an Eclectic School, "Eclectic Medical College of Pennsylvania," Prof. Jos. Sites, Dean, 514 Pine St., Phil. Both Schools regularly organized and chartered by the State Legislature, and honorable institutions. I refer you to the above-named gentlemen for any further information in regard to the Schools. Very respectfully,

A. J. HALE, M.D.

*214 Jacoby St., Phil.*

We think it quite evident that a systematic business of swindling, in the way of selling diplomas, has been carried on for some time in this country, with head-quarters at Philadelphia. The Milwaukee concern is probably only a branch. Is it not time that efficient steps were taken to put an end to this system of swindling?

### Mortality for the Month of December, 1869:—

| | |
|---|---|
| Accident, crushed | 1 |
| " by fall | 3 |
| " fracture of skull | 1 |
| " by machinery | 1 |
| " run over by street cars | 1 |
| " scalded | 2 |
| Abortion | 1 |
| Aneurism, aorta | 1 |
| Anasarca | 1 |
| Angina pectoris and biliary calculi | 1 |
| Apoplexy | 7 |
| Arachnitis | 2 |
| Ascitis | 1 |
| Births, still | 39 |
| " premature | 8 |
| " tedious | 1 |
| Bowels, inflammation | 1 |
| " ulceration | 1 |
| " inflammation, in con. of mucous hernia, | 1 |
| Brain congestion | 3 |
| " compression | 2 |
| " inflammation of | 1 |
| " effusion in, and paralysis | 1 |
| " softening of | 3 |
| Bronchitis | 6 |
| " and pleurisy | 1 |
| " chronic | 2 |
| " capillary | 1 |
| Catarrh | 1 |
| Cholera infantum | 1 |
| Convulsions | 47 |
| " puerperal | 1 |
| Consumption | 48 |
| " and syphilis | 1 |
| " fol'ng childbirth | 1 |
| Cyanosis | 1 |
| Croup | 25 |
| " diphtheretic | 2 |
| " membranous | 2 |
| Cyanche, maligna | 1 |
| Development deficient | 1 |
| Delirium tremens | 1 |
| " " and congestion of brain | 1 |
| Diarrhœa | 1 |
| " and convulsions | 1 |
| " chronic | 1 |
| Diphtheria | 14 |
| " and epilepsy | 1 |
| Dropsy | 3 |
| " and pericarditis | 1 |
| Dysentery | 1 |
| Encephalitis | 1 |
| Enteritis | 8 |
| Epilepsy and delirium tremens | 1 |
| Erysipelas | 4 |
| " of head and face | 1 |
| " and rheumatism | 1 |
| Fever, congestive | 2 |
| " puerperal | 5 |
| " congestion brain | 1 |
| " remittent | 3 |
| " scarlet | 44 |
| " " complications | 11 |
| " malignant | 4 |
| " typhoid | 9 |
| Gastritis | 2 |
| Groin, cancer of | 1 |
| Hemorrhage, internal | 1 |
| Heart, disease of | 1 |
| " paralysis of | 1 |
| " valvular disease | 2 |
| Hepatitis | 1 |
| Hydrocephalus | 8 |
| Inanition | 10 |
| Intemperance | 2 |
| " and exposure | 1 |
| Kidneys, Bright's disease of | 4 |
| " congestion of | 2 |
| Laryngitis | 5 |
| Liver, cancer of | 1 |
| " " and ascitis | 1 |
| " congestion of | 2 |
| " abscess of | 1 |
| Lungs, congestion of | 5 |
| " emphysema of | 1 |
| " hemorrhage of | 1 |
| Manslaughter | 2 |
| Measles | 2 |
| Meningitis | 3 |
| " cerebro-spinal | 2 |
| " tubercular | 4 |
| Old age | 5 |
| Ossification of ascending aorta | 1 |
| Ovarian tumor | 1 |
| Paralysis of nervous centres | 1 |
| Peritonitis | 2 |
| " & abscess psoas | 1 |
| " inflammation of uterus | 1 |
| " result of perforation of intest'ns | 1 |
| " following childbirth | 1 |
| Pleurisy | 1 |
| Pneumonia | 22 |
| Pyæmia | 1 |
| Rectum, prolapsus of | 1 |
| Scald-head | 1 |
| Scrofula | 2 |
| Spine, inflammation of | 1 |
| Stomach, cancer of | 2 |
| Suicide poisoning | 2 |
| " shooting | 2 |
| Syphilis | 1 |
| " hereditary | 1 |
| Tabes mesenterica, | 9 |
| Teething | 4 |
| Tetanus | 1 |
| Urine suppression of | 1 |
| Uræmia | 1 |
| Uterine hemorrhage | 1 |
| Uterus, cancer of | 2 |
| Whooping-cough | 3 |
| Unknown | 1 |
| Total | 438 |

COMPARISON.

Deaths in Dec., 1869, 438 | Deaths in Dec., 1868, 375 | Increase, 63
Deaths in Nov, 1869, 490 | Decrease, 52

AGES.

| | |
|---|---|
| Under 1 | 114 |
| 1 to 3 | 85 |
| 3 to 5 | 37 |
| 5 to 10 | 35 |
| 10 to 20 | 17 |
| 20 to 30 | 35 |
| 30 to 40 | 44 |
| 40 to 50 | 32 |
| 50 to 60 | 8 |
| 60 to 70 | 19 |
| 70 to 80 | 7 |
| 80 to 90 | 1 |
| 90 to 100 | 1 |
| Unknown | 3 |
| Total, | 438 |

| | | | | | |
|---|---|---|---|---|---|
| Males, | 253 | Females, | 185 | Total, | 438 |
| Single, | 320 | Married | 118 | Total, | 438 |
| White, | 434 | Colored, | 4 | Total, | 438 |

NATIVITY.

| | | | | | |
|---|---|---|---|---|---|
| Austria | 1 | England, | 8 | Sweden, | 9 |
| Bohemia, | 3 | France, | 2 | Scotland, | 3 |
| Canada, | 2 | Germany, | 65 | Wales | 3 |
| Chicago, Native, | 74 | Ireland, | 32 | Unknown, | 8 |
| Chicago, Foreign, | 151 | Norway, | 9 | | |
| U. S., other parts, | 64 | Russia | 1 | Total, | 438 |
| Denmark, | 3 | | | | |

MORTALITY BY WARDS FOR THE MONTH.

| Wards. | Mortality. | Pop. in 1868. | One death in |
|---|---|---|---|
| 1 | 7 | 9,094 | 1299 |
| 2 | 25 | 13,074 | 523 |
| 3 | 22 | 15,076 | 685¼ |
| 4 | 16 | 17,796 | 1112¼ |
| 5 | 27 | 16,033 | 593 4-5 |
| 6 | 24 | 13,083 | 541 |
| 7 | 28 | 25,492 | 910½ |
| 8 | 32 | 15,813 | 522⅓ |
| 9 | 30 | 19,297 | 643¼ |
| 10 | 11 | 12,925 | 1175 |
| 11 | 27 | 14,340 | 531 |
| 12 | 50 | 17,485 | 349 2-3 |
| 13 | 19 | 11,164 | 587½ |
| 14 | 23 | 14,839 | 645⅙ |
| 15 | 38 | 21,078 | 554 2-3 |
| 16 | 17 | 15,465 | 909 2-3 |

| Mortality. | |
|---|---|
| Accidents, | 9 |
| County Hospital, | 13 |
| Immigrants, | 1 |
| Manslaughter, | 2 |
| Mercy Hospital, | 2 |
| St. Joseph Orphan Asylum, | 9 |
| Soldier's Home, | 1 |
| Suicide, | 4 |
| Jewish Hospital, | 1 |
| Total, | 438 |

